# Ozone Gel in Chronic Periodontal Disease: A Randomized Clinical Trial on the Anti-Inflammatory Effects of Ozone Application

**DOI:** 10.3390/biology10070625

**Published:** 2021-07-06

**Authors:** Marco Colombo, Simone Gallo, Alessandro Garofoli, Claudio Poggio, Carla Renata Arciola, Andrea Scribante

**Affiliations:** 1 Section of Dentistry, Department of Clinical, Surgical, Diagnostic and Paediatric Sciences, University of Pavia, Piazzale Golgi 2, 27100 Pavia, Italy; marco.colombo@unipv.it (M.C.); alessandro.garofoli01@universitadipavia.it (A.G.); andrea.scribante@unipv.it (A.S.); 2 Laboratorio di Patologia delle Infezioni Associate all’Impianto, IRCCS Istituto Ortopedico Rizzoli, Via di Barbiano 1/10, 40136 Bologna, Italy; 3 Department of Experimental, Diagnostic and Specialty Medicine, University of Bologna, Via San Giacomo 14, 40136 Bologna, Italy

**Keywords:** dentistry, periodontitis, scaling and root planing, ozone, chlorhexidine, clinical trial, anti-infective, implant infections, antibiotic resistance

## Abstract

**Simple Summary:**

The search for new topical antimicrobial treatments represents an actual challenge both in orthopedics and in dentistry. As regards the latter, antiseptics represent an aid to the non-surgical treatment commonly employed to contrast periodontitis. This study aims to assess the efficacy of an ozone-based gel with respect to the more common chlorhexidine gels. Ten participants were treated by means of nonsurgical periodontal therapy, with, in addition, a chlorhexidine gel and an ozone one, both, respectively, used in half of the oral sites. Patients were visited at baseline, after one month, and after three months, and at each time point clinical indexes were assessed. This study revealed that the use of the ozonized gel in addition to the standard non-surgical therapy generally did not significantly differ if compared to the use of chlorhexidine. Based on this, ozone deserves consideration for its wide applicability in several clinical fields, especially considering the reduced number of shortcomings with respect to those generally related to chlorhexidine.

**Abstract:**

The search for new topical treatments able to display not only antimicrobial properties but also a multiplicity of other beneficial effects while expressing safe cytocompatibility toward host tissues is being progressively developed. Antiseptics represent an aid to the gold standard nonsurgical treatment Scaling-and-Root-Planing (SRP) for periodontal disease. This split-mouth study aims to assess the efficacy of the ozonized gel GeliO_3_ (Bioemmei Srl, Vicenza, Italy) plus SRP (experimental treatment), with respect to SRP + chlorhexidine gel. Ten participants were treated with SRP + chlorhexidine gel (control sites) and with SRP + ozone gel (trial sites). After 1 (T_1_) and 3 months (T_2_) from baseline (T_0_), patients were revisited. At each time-point, the following indexes were assessed: probing pocket depth (PPD), clinical attachment level (CAL), gingival index (GI), plaque index (PI), and bleeding on probing (BoP). It has been assessed that the use of the ozonized gel in addition to SRP did not show significant differences if compared to conventional SRP + chlorhexidine. Chlorhexidine was found to be more effective than ozone in reducing CAL and GI at T_2_. Ozone deserves consideration for its wide applicability in several clinical fields. In this connection, we also glance at the latest research on ozone therapy.

## 1. Introduction

Over the last few years, the use of ozone in medicine has significantly raised due to its recognized properties. Several in vitro studies have shown wide antibacterial activity for ozonized vegetable oils against microorganisms, such as bacteria, virus, protozoa, and fungi [1,2]. In addition to that, ozone shows immunomodulatory, anti-hypoxic, biosynthetic, and anti-inflammatory properties which justifies its several applications, both in orthopedics and dentistry [3]. As regards the latter, ozone therapy has been used to manage wounds healing, dental caries, oral lichen planus, gingivitis and periodontitis, halitosis, osteonecrosis of the jaw, post-surgical pain, plaque and biofilms, root canal treatment, dentin hypersensitivity, temporomandibular joint disorders, and teeth whitening [3,4], and to functionalize implants surfaces for dental and orthopedic clinical uses [3]. Considering the abovementioned applications, the use of ozone for the treatment of gingivitis and periodontitis appears quite interesting for clinicians. In particular, this latter condition is not only more and more frequent among patients but, in some cases, might be refractory to the treatment. Gingivitis arises from the accumulation on the teeth of dental plaque, corresponding to a complex biofilm of bacteria dipped into a polymeric matrix. If this biofilm is not properly removed by means of oral hygiene, gingivitis might develop into periodontitis, with a destruction of tooth-supporting tissues, in presence of other predisposing factors among which smoke, diabetes, immune disorders, etc. [5]. 

Scaling and root planing (SRP) is a gold standard non-surgical therapy, which is aimed both to remove dental plaque and calculus as well as to smooth the root surfaces infected by bacteria [6]. In addition to mechanical therapy, local chemical treatments should be favored in comparison to systemic treatments like antibiotics which are linked to the risk of emergence of resistance. In this context, the use of ozone as an antimicrobial could be very useful considering the pathogenetic action exerted by bacteria in the development and maintaining of periodontal inflammation. Broad spectrum antiseptics such as chlorhexidine are often used, but toxicological aspects should be considered. In contrast, ozone has also a broad spectrum of activity but with low toxicity, giving an advantage to this local treatment before the development of substances with a more specific action on the micro-organisms related to periodontitis. 

In vitro exposition of bacteria to ozone causes the oxidation of phospholipids and lipoproteins constituting the bacterial cell envelope; this event leads to the disruption of the cytosolic membrane integrity, thus allowing ozone to infiltrate the microorganisms and oxidize glycoproteins and glycolipids, with a final block of the bacterial enzymatic function [3]. 

The aim of the present study is to evaluate the efficacy of the subgingival application of an experimental ozone gel in addition to standard SRP, as well as to compare this protocol with SRP plus a conventional chlorhexidine gel. To the best of our knowledge, few studies in literature have compared, so far, the efficacy of ozone with respect to chlorhexidine in periodontal nonsurgical therapy. The null hypothesis of the study is that there are no significant intergroup and intragroup differences between the two different oral gels.

## 2. Materials and Methods

### 2.1. Material

The products used for the experimentation and their characteristics are shown in Table 1. 

### 2.2. Trial Design

This study has been designed as a prospective single-group and single-center randomized clinical trial which has been approved by the Internal Review Board (number of approval: 2020-0708). No changes to the methods occurred after the commencement of the study. According to previous research, this study was designed as a split mouth study with the subdivision of the mouth into quadrants [7]. 

The CONSORT document includes a checklist of 22 points which, from the Title to the Discussion, define the essential requirements for the presentation of a randomized clinical trial. In our paper we have referred to the points of the CONSORT document indicating how we have complied with them. The number of cases here studied is small but in compliance with the sample size calculation requested by the CONSORT document criteria. 

### 2.3. Participants, Eligibility Criteria, and Settings

Participants signed an informed consent to take part to the experimentation and to allow the publication of the results obtained. Starting from September 2020 until November 2020, 10 patients with periodontal disease were recruited at the affiliation of the Authors where all the experimental phases have taken place until the end of the study (February 2021). Selected participants of both sexes had to show periodontitis at stage III and grade B, according to the most recent classification of periodontal disease of (2018) [8]. 

People were excluded in case of the following situations: systemic diseases (e.g., uncontrolled diabetes, anemia, cardiovascular diseases, infectious diseases), systemic diseases-related periodontitis, pathologic conditions of the oral mucosa, presence of fixed prostheses and orthodontic appliances, untreated decays, use of chewing tobacco, smokers, alcoholics, treatment with chlorhexidine in the last 6 weeks, pregnancy or feeding, use of systemic drugs in the last 3 months (antibiotics, FANS, steroids, inhibitors of the salivary flow, and anticoagulant/immunostimulant/immunosuppressive/antimycotic drugs) and contemporary use of topical drugs for the oral cavity. Additionally, people were excluded in case of concomitant participation to other clinical trials or lack of telephone contact. 

### 2.4. Interventions and Outcomes

During the first visit, participants underwent a professional oral hygiene and chairside instructions were also given at this appointment.

Two weeks later participants underwent another appointment (considered as baseline) in which the following clinical indexes were assessed: probing pocket depth (PPD) (distance from the gingival margin to the pocket base), clinical attachment level (CAL) (differences between the position of the soft tissue in relation to the cement-enamel junction), gingival index (GI, Löe and Silness) (index 1–3, proportional to gingival inflammation), plaque index (PI—O’Leary) (percentage of sites with plaque) and bleeding on probing (BoP—Ainamo and Bay) (percentage of sites showing bleeding on probing) [5,9]. The two former indexes were, respectively. assessed on four sites for each tooth (mesial, buccal, distal and lingual) by means of a dental probe (UNC probe 15, Hu-Friedy, Chicago, IL, USA), whereas the other three indexes were measured on six sites (mesio-buccal, buccal, disto-buccal, mesio-lingual, lingual and disto-lingual). 

At this same appointment, patients were allocated to the respective treatment. Each quadrant of the mouth of the participants was randomly assigned to a treatment with SRP + chlorhexidine gel (control sites) and with SRP + ozone gel (trial sites), according to split mouth design. Only two or all four quadrants of each patient’s mouth were treated, depending on the number of sites with periodontal disease. SRP was conducted using a piezoelectric (Mini Piezon, EMS; Nyon, Switzerland) and Gracey curettes (Hu-Friedy, Chicago, IL, USA), whereas the treatment with the oral gels consisted of a subgingival application by means of a syringe. 

After 1 (T_1_) and 3 months (T_2_) from baseline (T_0_), patients were revisited; in case of necessity, a further professional supragingival oral hygiene was conducted at these appointments. In addition to that, periodontal clinical indexes were assessed again as previously described. 

Chairside instructions for a correct domiciliary oral hygiene were repeated to participants at each appointment.

No changes to the trial outcomes occurred after the trial commencement. 

### 2.5. Sample Size Calculation

Sample size calculation (Alpha = 0.05; Power = 90%) for an independent study group and a continuous primary endpoint was performed. As regards the variable gingival index, an expected mean of 1.80 was hypothesized, with a standard deviation of 0.60 [10]. This variable was chosen as primary outcome, according to the studies considered in the recent systematic review with meta-analysis by Akram et al. [11]. The expected difference between the means was supposed to be 1.2, therefore 10 patients were requested. Loss to follow-up and incomplete compliance with therapy were excluded.

A total of 10 patients were visited before the trial commencement and then selected for the study according to the sample size calculation. No one refused to participate or did not meet the inclusion criteria. 

On sampling, no distinction between anterior and posterior teeth has been considered, as with previous similar studies [12,13]. 

### 2.6. Randomization, Sequence Generation, Allocation Concealment and Implementation

Participants were randomized to their respective group using a random number table. Two permuted blocks of five patients each were considered. Allocation concealment was achieved with sequentially numbered, opaque, sealed envelopes containing the treatment allocation cards which had been prepared before the trial.

The generation of the random allocation sequence, the enrollment of participants and the assignment to interventions were performed by three different clinicians not further involved in the study, in order to avoid bias. 

### 2.7. Blinding

Professional oral procedures and outcomes assessment were respectively executed by two operators. Blinding the operator administering the treatment assigned was not technically possible but this one was not involved in any other phase of the study and was not in contact with the other researchers. Conversely, the data assessor and data analyst were always blinded during the study since none of them knew the treatment administered to each participant. Patients were asked not to reveal their respective treatment to the data assessor.

### 2.8. Statistical Methods

Data were submitted to statistical analysis with R Software (R version 3.1.3, R Development Core 150 Team, R Foundation for Statistical Computing, Wien, Austria). For each variable, descriptive statistics (mean, standard deviation, median, minimum and maximum value) were calculated. PPD and CAL were measured in millimeters (mm), whereas PI and BoP were measured as percentages and GI was measured with the relative score (0–3). 

Data normality was calculated using the Kolmogorov–Smirnov test and subsequently a Student’s *t*-test was applied. Significance for all statistical tests was predetermined at *p* < 0.05.

## 3. Results

### 3.1. Participant Flow

**Figure 1:**
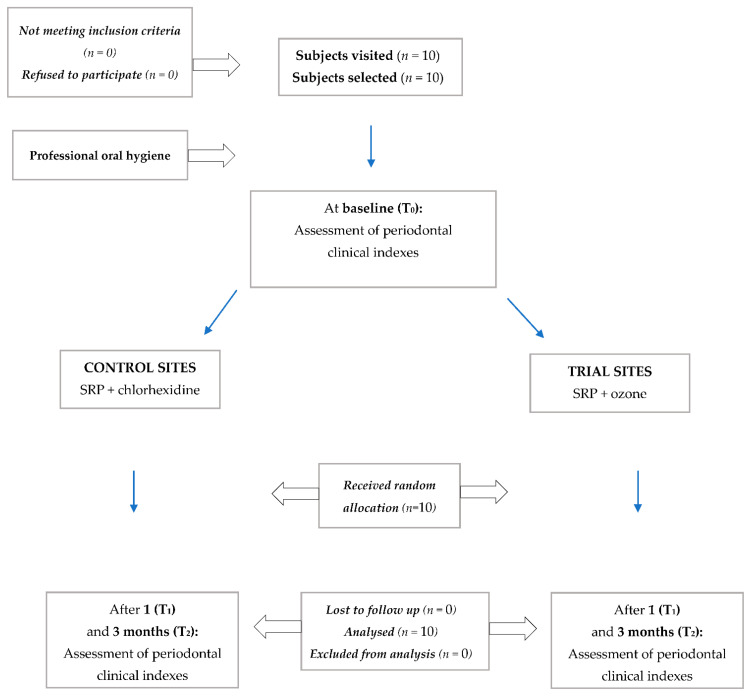
Flow-chart of the study.

The flow chart of the study is shown in Figure 1. The quadrants of the mouth of 10 patients (4 males and 6 females, mean age 50 years old) were randomized in a 1:1 ratio. No patient refused to participate and so they were all assigned to the respective treatment. There was no loss to follow up or exclusion from analysis. 

### 3.2. Recruitment

Participants were recruited from September 2020 until November 2020. According to the 3-month follow up, the study ended in February 2021. The trial neither ended nor was stopped in advance.

### 3.3. Baseline Data

Patients recruited in the study consisted of 4 males and 6 females, with middle age of 50 y.o. The baseline clinical characteristics of the patients are reported in Table 2, divided considering the trial and control sites. 

### 3.4. Numbers Analyzed

All patients randomized have been included in each analysis. 

### 3.5. Outcomes and Estimation

The descriptive statistics of the clinical indexes assessed are shown in Table 3.

#### 3.5.1. Probing Pocket Depth (PPD)

Significant intragroup differences were found between each time point both for the sites treated with SRP plus ozone and for the sites treated with SRP plus chlorhexidine (*p* < 0.05); no significant intergroup differences were found between the sites (*p* > 0.05) (Table 3).

Specifically, as regards the reduction of PPD in both trial and control sites from T_0_ to T_2_, data are shown in Table 4. 

#### 3.5.2. Clinical Attachment Level (CAL)

Significant intragroup differences were found between each time point for the sites treated with SRP plus chlorhexidine (*p* < 0.05), whereas for ozone a significant improvement was found at T_1_ but not at T_2_; a significant intergroup difference was found between the sites at T_2_ (*p* < 0.05) (Table 3).

#### 3.5.3. Gingival Index (GI)

Significant intragroup differences were found between each time point both for the sites treated with SRP and ozone (but not comparing T_1_-T_2_) and for the sites treated with SRP plus chlorhexidine (*p* < 0.05); a significant intergroup difference was found between the sites at T_2_ (*p* < 0.05) (Table 3).

#### 3.5.4. Plaque Index (PI)

Significant intragroup differences were found between each time point both for the sites treated with SRP plus ozone and for the sites treated with SRP plus chlorhexidine (*p* < 0.05); no significant intergroup differences were found between the sites (*p* > 0.05) (Table 3).

#### 3.5.5. Bleeding on Probing (BoP)

Significant intragroup differences were found between each time point both for the sites treated with SRP plus ozone and for the sites treated with SRP plus chlorhexidine (*p* < 0.05); no significant intergroup differences were found between the sites (*p* > 0.05) (Table 3).

### 3.6. Ancillary Analyses

No other analyses have been performed.

### 3.7. Harms

No harms have been observed for the treatment assigned to each group.

## 4. Discussion

Scaling and root planing (SRP) represents the gold standard therapy for the treatment of periodontal disease, along with the concomitant use of antibiotics and/or antiseptics [5,14]. In order to propose new chemical compounds, the major goal of the present study was to assess the efficacy of subgingival applications of ozone gel in addition to SRP, with respect to SRP plus a conventional chlorhexidine gel. Intergroup and intragroup differences at the various times have been conducted in order to assess which chemical compound could be more beneficial for the treatment of periodontitis in addition to SRP. The null hypotheses of the study were that no significant intergroup and intragroup differences occur between the experimental treatment and the control one, which were both partially refused. 

Our results show that all clinical indexes tested (Probing pocket depth, PPD; Clinical Attachment Level, CAL; Gingival Index, GI; Plaque Index, PI; and Bleeding on Probing, BoP) significantly improved after 1 and 3 months, with respect to the baseline. This tendency was confirmed both for the experimental and the control condition in the split mouth study design considered. In addition to that, intragroup differences were generally significant, differently from intergroup differences. According to these results, the experimental protocol combining ozone to conventional SRP seems to be a reliable option for the nonsurgical management of the periodontal disease. The improvement of all the clinical indexes following the treatment with SRP plus subgingival applications of ozone might be due to the antimicrobial effects of this latter being an oxidant [15]. However, this chemical compound can also induce the release of growth factors, cause a vascular and hematological modulation, stimulate the immune system, and activate local antioxidant mechanisms if administered at low doses [16,17]. In particular, despite no intragroup differences with chlorhexidine occur at any time for BoP, the reduction of this index in the quadrants treated with GeliO_3_ might be due not only to an antimicrobial effect (as also happens for chlorhexidine) but especially to the anti-inflammatory and antioxidant action.

Conversely, focusing on the significant improvement for PPD and CAL, this is due to the repair of connective tissue, ascribable to the stimulating action of ozone towards fibroblasts, but also to an increase angiogenesis with revascularization of the gingival tissue [18].

In this study, no significant intergroup differences were assessed for most of the indexes assessed. According to this outcome, despite the valuable effect of ozone in addition to SRP, the results obtained after 1 and 3 months were generally the same assessed for SRP plus chlorhexidine. Additionally, chlorhexidine was more effective than ozone in reducing CAL and GI at T_2_, and there was no difference between chlorhexidine and ozone in T3.

Previous studies in literature were carried out to compare the efficacy of ozonized and chlorhexidine-based products to deal with periodontitis. Most of these reports generally agree with our results by showing no statistical differences between the two antiseptics or a slightly better improvement for ozone with respect to chlorhexidine [19]. In other cases, greater outcomes have been described for ozone therapy [20]. The results of the systematic review by Moraschini et al. [21] do not support the use of ozone therapy for nonsurgical periodontal treatment. 

The current evidence indicates that ozone has antimicrobial activity and good biocompatibility with periodontal cells and gingival fibroblasts [22,23]. However, no evidence was found for a positive effect of ozone therapy as an adjunct to scaling and root planing.

According to the results reported in this study, the use of the ozonized gel GeliO_3_ inside a protocol for the non-surgical management of periodontal disease represents a valid approach, even if without a greater effect with respect to the standard SRP plus chlorhexidine. However, it should be considered that chlorhexidine exhibits higher cytotoxic effects, which might be a valid reason to prefer the use of ozone in non-surgical periodontal therapy instead of the former [3,24,25,26]. Although the use of chlorhexidine is cheaper, the recourse to ozone in the dental clinic with ozone generators could be justified, also considering the broad spectrum of beneficial effects of the latter substance.

The mouth houses a diverse symbiotic microbiota organized in biofilms that colonize the mucous membranes and dental surfaces. The oral microbiota exerts beneficial effects on the host, as it resists and counteracts colonization by pathogenic microorganisms, (b) attenuates the host’s inflammatory responses and (c) participates in the physiological development of the immune defenses of the mouth. Under pathological conditions, this harmonious symbiotic relationship fails and a condition of dysbiosis occurs. In dysbiosis, the proportion of different bacterial species changes with the transition to a higher prevalence of anaerobic and proteolytic species endowed with high destructive potential. They can damage tissues and cause diseases of the teeth and mouth, such as tooth decay, periodontitis, pocket formation and loss of attachment [27]. 

Ozone is a broad-spectrum antimicrobial agent (like chlorhexidine), which proved to be able to reduce the periodontitis bacterial burden. Moreover, as mentioned above, ozone appears to be worthy of particular consideration for its low toxicity compared to chlorhexidine [28]. New agents more specifically active on periodontopathogens are now emerging and are being studied, such as Oxysafe [29].

The study model and the results obtained could have a utility that goes beyond the dental field. Indeed, the oral cavity lends itself to being observed and the lesions of the buccal mucosa can be followed in their evolution. Thus, they could be somewhat representative of the response of other similar tissues, such as mucosae other than oral. By controlling and resolving periodontitis, the imbalance of oral microbiota is countered. This should help defuse and extinguish those conditions of chronic inflammation that can favor the onset of more general diseases. 

On the other hand, a major limitation of this study is that only clinical parameters have been tested. It would be interesting to even perform microbiological tests to compare in vitro the antimicrobial action of the two products tested. In addition to that, further randomized clinical trials should be performed to evaluate a longer follow up in order to verify whether a long-term effect can be guaranteed as well. Finally, as to the external validity of the results, the efficacy of chlorhexidine and ozone cannot be directly generalized to other stages/grades of the pathology with respect to those considered as inclusion criteria in this trial, except at the level of hypotheses and speculations.

**Figure 2:**
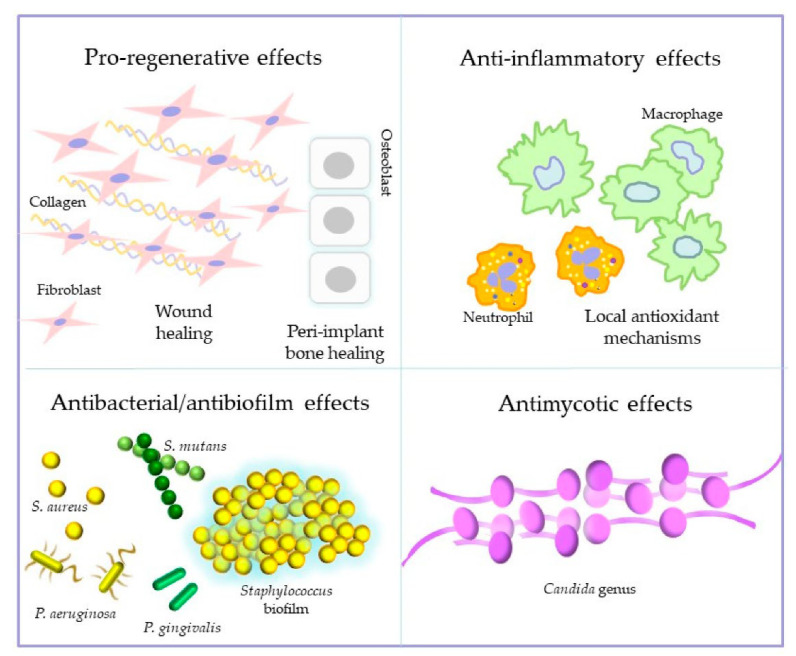
Principal biological effects reported for ozone treatments.

Although these findings relate to the specific aim of this research, they provide some food for thought for more general reflection. In the last few years, a renewed interest in the therapeutic potential of ozone has emerged. In particular, the focus is on the ability to promote wound healing, to attenuate the adverse effects of inflammation by reducing the oxidative activities of inflammatory cells, to express antimicrobial activities against various bacterial species and mycetes pathogenic for humans, and also against biofilm-producing and antibiotic-resistant bacteria, thus offering chances of overcoming antibiotic-resistance issues [30,31,32,33,34,35,36,37,38,39] (Figure 2). 

Interestingly, some studies show that ozone treatments could be useful in combating implant-associated infections, exhibiting antibacterial activity and promoting osseointegration [32,33]. Furthermore, ozone-functionalized dental and orthopedic implant materials seem to favorably influence the behavior of bone marrow cells and macrophages [34]. 

Ozone is also considered attractive for veterinary and food applications, which is important in the new era of the holistic “one health” view [40,41]. 

## 5. Conclusions

In addition to SRP, the use of the ozonized gel GeliO_3_ can be regarded as a valid substitute to chlorhexidine, especially considering the major shortcomings associated with the latter.

## Figures and Tables

**Table 1:** Products tested in the study.

Product	Description	Ingredients	Manufacturer
GeliO_3_	Ozonized gel	Bio-ozonized olive oil (20 mEq O_2_/Kg), Hydrated Silica, Arnica	Bioemmei Srl, 36100 Vicenza, Italy
Curasept Parodontal gel 1% Ads	Chlorhexidine gel	Sorbitol, Aqua, Hydrated Silica, Glycerin, Xylitol, PEG-40 Hydrogenated Castor Oil, Cocamidopropyl Betaine, Aroma, Cellulose Gum, Chlorhexidine, Digluconate, Ascorbic Acid, Sodium Metabisulfite, Sodium Saccharin, Sodium Methylparaben, Sodium Citrate, CI 42090	Curasept SPA, 21047 Saronno, Varese, Italy

**Table 2:** Baseline characteristics of patients.

Clinical Indexes	Control Sites (Mean ± SD)	Trial Sites (Mean ± SD)
PPD (mm)	5.94 ± 0.89	6.21 ± 0.92
CAL (mm)	6.13 ± 0.81	6.00 ± 0.83
GI (0–3)	1.67 ± 0.39	1.67 ± 0.56
PI (%)	0.86 ± 0.16	0.85 ± 0.18
BOP (%)	Cs: 0.33 ± 0.13	0.43 ± 0.27

SD: standard deviation.

**Table 3:** Descriptive statistics (mean, SD, minimum value, median, maximum value) of the clinical indexes assessed in the study: PPD, CAL, GI, PI, BOP.

Clinical Index	Treatment	Time	Mean	SD	Min	Median	Max	Intragroup Differences *	Intergroup Differences *
PPD (mm)	SRP + Ozone	T_0_	6.21	0.92	5.25	6.27	8.40	T_0_-T_1_ *p* < 0.05	T_0_-T_0_ > 0.05 T_1_-T_1_ > 0.05 T_2_-T_2_ > 0.05
		T_1_	4.66	0.74	3.83	4.48	5.50	T_0_-T_2_ *p* < 0.05	
		T_2_	4.20	0.48	3.50	4.15	5.20	T_1_-T_2_ *p* > 0.05	
	SRP + Chlorhexidine	T_0_	5.94	0.89	5.00	5.89	7.50	T_0_-T_1_ *p* < 0.05	
		T_1_	4.42	0.76	3.37	4.35	5.78	T_0_-T_2_ *p* < 0.05	
		T_2_	3.95	0.52	3.25	3.90	4.95	T_1_-T_2_ *p* > 0.05	
CAL (mm)	SRP + Ozone	T_0_	6.00	0.83	5.10	5.89	7.50	T_0_-T_1_ *p* < 0.05	T_0_-T_0_ *p* > 0.05 T_1_-T_1_ *p* > 0.05 T_2_-T_2_ *p* < 0.05
		T_1_	4.42	0.76	3.37	4.35	5.78	T_0_-T_2_ *p* < 0.05	
		T_2_	4.32	0.47	3.45	4.32	4.94	T_1_-T_2_ *p* > 0.05	
	SRP + Chlorhexidine	T_0_	6.13	0.81	5.25	6.06	8.00	T_0_-T_1_ *p* < 0.05	
		T_1_	4.85	0.90	3.83	4.54	6.50	T_0_-T_2_ *p* < 0.05	
		T_2_	3.99	0.56	3.15	4.05	4.91	T_1_-T_2_ *p* < 0.05	
GI (1–3)	SRP + Ozone	T_0_	1.67	0.56	0.80	1.54	2.56	T_0_-T_1_ *p* < 0.05	T_0_-T_0_ *p* > 0.05 T_1_-T_1_ *p* > 0.05 T_2_-T_2_ *p* < 0.05
		T_1_	1.01	0.38	0.50	0.95	1.60	T_0_-T_2_ *p* < 0.05	
		T_2_	0.91	0.35	0.37	0.93	1.45	T_1_-T_2_ *p* > 0.05	
		T_0_	1.67	0.39	0.87	1.80	2.14	T_0_-T_1_ *p* < 0.05	
	SRP + Chlorhexidine	T_1_	1.06	0.38	0.45	1.15	1.50	T_0_-T_2_ *p* < 0.05	
		T_2_	0.71	0.36	0.05	0.75	1.30	T_1_-T_2_ *p* < 0.05	
PI (%)		T_0_	85	18	55	90	100	T_0_-T_1_ *p* < 0.05	T_0_-T_0_ *p* > 0.05 T_1_-T_1_ *p* > 0.05 T_2_-T_2_ *p* > 0.05
	SRP + Ozone	T_1_	54	9	40	50	70	T_0_-T_2_ *p* < 0.05	
		T_2_	39	7	27	40	50	T_1_-T_2_ *p* < 0.05	
	SRP + Chlorhexidine	T_0_	86	16	60	90	100	T_0_-T_1_ *p* < 0.05	
		T_1_	52	7	40	50	65	T_0_-T_2_ *p* < 0.05	
		T_2_	36	8	25	34	50	T_1_-T_2_ *p* < 0.05	
BOP (%)		T_0_	43	27	7	40	87	T_0_-T_1_ *p* < 0.05	T_0_-T_0_ *p* > 0.05 T_1_-T_1_ *p* > 0.05 T_2_-T_2_ *p* > 0.05
	SRP + Ozone	T_1_	15	6	5	17	24	T_0_-T_2_ *p* < 0.05	
		T_2_	9	4	2	9	15	T_1_-T_2_ *p* < 0.05	
	SRP + Chlorhexidine	T_0_	33	13	18	31	50	T_0_-T_1_ *p* < 0.05	
		T_1_	11	7	2	10	24	T_0_-T_2_ *p* < 0.05	
		T_2_	9	6	2	7	17	T_1_-T_2_ *p* < 0.05	

* Significant differences for *p* < 0.05. SD: standard deviation.

**Table 4:** Reduction of PPD in trial and control sites from baseline to the end of the study.

PPD (mm)	T_0_	T_2_	T_0_-T_2_	SD
SRP + Ozone	6.21	4.20	2.01	1.42
SRP + Chlorhexidine	5.94	3.95	1.99	1.41

SD: standard deviation.

## Data Availability

The data presented in this study are available on request from the corresponding authors.
